# Epidemiology of non-communicable diseases among professional drivers in LMICs: a systematic review and meta-analysis

**DOI:** 10.1093/heapro/daae087

**Published:** 2024-08-31

**Authors:** Belinda J Njiro, Harrieth P Ndumwa, Hannah Wanjiku Waithera, Rehema Chande, William Julius, Fredirick Mashili, Julius C Mwita, Monica H Swahn, Catherine Staton, Joel Msafiri Francis

**Affiliations:** MRC/Wits Rural Public Health and Health Transitions Research Unit (Agincourt), Faculty of Health Sciences, School of Public Health, University of the Witwatersrand, Johannesburg, South Africa; Department of Global Public Health and Primary Care, University of Bergen, Bergen, Norway; Division of Epidemiology and Biostatistics, School of Public Health, University of the Witwatersrand, Johannesburg, South Africa; Directorate of Library Services, Muhimbili University of Health and Allied Sciences, Dar es Salaam, Tanzania; Directorate of Library Services, Muhimbili University of Health and Allied Sciences, Dar es Salaam, Tanzania; Department of Physiology, School of Biomedical Sciences, Muhimbili University of Health and Allied Sciences, Dar es Salaam, Tanzania; Department of Internal Medicine, University of Botswana and Princess Marina Hospital, Gaborone, Botswana; Wellstar College of Health and Human Services, Kennesaw State University, Kennesaw, GA, USA; Department of Emergency Medicine, Duke School of Medicine/Duke Global Health Institute, Duke University, Durham, NC, USA; Department of Family Medicine and Primary Care, University of the Witwatersrand, Johannesburg, South Africa

**Keywords:** non-communicable diseases, hypertension, diabetes mellitus, obesity, professional drivers, low and middle-income countries

## Abstract

This systematic review collected evidence on the burden of non-communicable diseases (NCDs) among professional drivers and reported on the most common factors that increase the risk of NCDs in this specific population in low- and middle-income countries (LMICs). The protocol for this systematic review was registered in the International Prospective Register of Systematic Reviews (PROSPERO). We conducted a thorough search on PubMed/MEDLINE, EMBASE, Scopus, Global Health, Web of Science and Africa-wide information databases on 11 May 2023. We adapted the Joanna Briggs Institute (JBI) tool to assess the quality of the studies. We estimated the prevalence of hypertension, prediabetes, diabetes mellitus (DM), overweight and obesity among professional drivers using a random effect model to compute pooled and subgroup analyses. In addition, we conducted a narrative synthesis of the risk factors and recommendations presented in the included studies. Forty-one studies, including 48 414 study participants, met the criteria for inclusion. The pooled prevalence of hypertension, DM and obesity among professional drivers was 36.7% [95% confidence interval (CI): 31.8–41.6%], 15.2% (95% CI: 7.0–23.4%) and 27.2% (95% CI: 18.7–35.8%), respectively. Unsupportive environment, work stress, sedentary lifestyle, consumption of unhealthy foods and shift work were the most common modifiable risk factors reported. Our findings also show a significant burden of hypertension, DM and obesity among professional drivers in LMICs. The prevalence of DM and obesity was two- and three-fold higher than findings in general populations, respectively. Our findings indicate an urgent need for tailored interventions for different occupation-related risk factors for NCDs among professional drivers in LMICs.

**Contribution to Health Promotion**

- Professional drivers in low- and middle-income countries (LMICs) and elsewhere are at increased risk of non-communicable diseases (NCDs) due to physical inactivity, sleep deprivation and poor eating habits as a result of unique working conditions, including long hours of sitting during working hours.
- Our review, based on 41 studies, including 48 414 participants representing professional drivers in LMICs, finds a high prevalence of three common NCDs (hypertension 36.7%, diabetes 15.2% and obesity 27.2%), indicating an urgent need for targeted interventions in this understudied but high-risk population.
- Health promotion efforts for this population should include educational programs, screenings for NCDs, enhanced workplace policies and provision of facilities for physical exercise.

## BACKGROUND

A consistent link exists between occupational factors, physical inactivity and non-communicable diseases (NCDs) (Wahid *et al*., 2016). Compared to the general public, professional drivers are at increased risk of NCDs due to physical inactivity and a sedentary lifestyle from long hours of sitting during distant travels (Udayar *et al*., 2015), unhealthy diet, obesity (Yosef *et al*., 2020), harmful alcohol intake and tobacco (Udayar *et al*., 2015; Rao *et al*., 2016). Moreover, with work-related stress and tension (Mohsen and Hakim, 2019), changes in the sleep–wake cycle and sleep deprivation further increase their NCD morbidity and mortality risk (Winkleby *et al*., 1988).

Following increased urbanization and lifestyle changes, there is a transition in the burden of diseases from communicable diseases to NCDs (Bigna and Noubiap, 2019). NCDs are currently the leading cause of mortality, with a total of 41 million deaths each year, accounting for 71% of all deaths globally. Cardiovascular diseases (CVDs), cancers, respiratory diseases and diabetes are the four top killers of NCDs, accounting for 80% of all NCD-related deaths (Forouzanfar *et al*., 2016). In low- and middle-income countries (LMICs), NCDs are responsible for even more disability-adjusted life years (Arun and Meriton Stanly, 2022). The commonest factors contributing to the rising burden of NCDs are raised blood pressure, tobacco smoking, harmful alcohol consumption, physical inactivity, raised blood glucose and obesity (Holt *et al*., 2013). Other factors include dietary habits and environmental pollution, as well as the role of genetics (Hamra *et al*., 2014; Peters *et al*., 2018). These may act individually or in combination to exacerbate risk. A significant link also exists between occupational factors that are associated with a sedentary lifestyle and physical inactivity and NCDs (Saunders *et al*., 2020), which may represent key concerns for professional drivers. Research demonstrates that professional drivers across various settings are experiencing an increasing burden of hypertension- and diabetes mellitus (DM)-related deaths (Odeyinka and Ajayi, 2017), which warrants urgent attention.

Occupation and working conditions have a significant role in health promotion and in the development of NCDs, but these have not frequently been addressed in LMICs. As a significant number of individuals spend most of their waking time at work, the workplace serves to influence health risks and outcomes globally (Largo-Wight *et al*., 2011; International Labour Office, 2012). The increasing burden of NCDs among sedentary workers, including professional drivers, has been reported in a diverse population due to the nature of their work (Rao *et al*., 2016).

In this paper, professional drivers refer to any individual who drives motor vehicles for a living, including long-haul drivers who are also faced with myriad other factors that predispose them to the double burden of communicable diseases and NCDs. Due to engagement in their work and their working conditions, professional drivers exercise less and have less access to healthcare services. This further increases the gender disparities in healthcare access as males make up the majority of this population (Solomon *et al*., 2004). In addition to the previously reported high-risk behaviours such as substance use that increased their risk of NCDs, the availability of healthy foods during trips is also a key factor contributing to poor dietary choices and eating behaviours (Bschaden *et al*., 2019). In India, harmful alcohol use and tobacco smoking were reported in 46% and 35% of drivers, respectively; 42% and 22% were obese and hypertensive, respectively (Girish *et al*., 2016).

Strategies and interventions for health promotion, prevention and control of NCDs are diverse and require a multisectoral approach beyond the healthcare system (Alwan and MacLean, 2009). With the double burden of diseases in LMICs, the availability and implementation of screening and management services for hypertension, DM and obesity is a challenge, especially among resource-constraint countries (Alwan and MacLean, 2009) where the burden of NCDs is increasing currently, and interventions and occupational health strategies are typically lacking. For example, professional drivers are presented with a unique working environment with minimal workplace health programmes available to address their NCD risk or other health concerns. Moreover, to the best of our knowledge, there is no collated evidence to guide tailored strategies and policies to address the risk of NCDs among professional drivers in LMICs. Such interventions should extend to address the difficulty they face in accessing healthcare services. We aimed to collate evidence on the burden of hypertension, DM and obesity among professional drivers and reported diverse and modifiable factors increasing the risk of NCDs among this sedentary population.

## METHODS

### Study design

We developed a systematic review protocol according to the Preferred Reporting Items for Systematic Reviews and Meta-Analyses Protocols (PRISMA-P) (Moher *et al*., 2009) before conducting this review. The protocol was registered in the International Prospective Register of Systematic Reviews (PROSPERO) database, registration number CRD42022380446.

### Eligibility criteria

We included only observational studies (cross-sectional, case–control and cohort studies) conducted among professional or commercial drivers diagnosed with the selected NCDs in LMICs. The targeted NCDs include CVDs, hypertension, DM and obesity. We excluded articles not reported in English; systematic reviews and meta-analyses were also excluded at full-text screening; however, we searched the reference list for relevant articles. No publication time limit was applied.

### Search strategy

We systematically searched articles from PubMed/MEDLINE, EMBASE, Scopus, Global Health, Web of Science and Africa-wide information on the 10th and 11th of May 2023. The search included studies conducted in human beings and published in English, with no allocated time limit. We developed a rigorous systematic search strategy with the help of a health sciences librarian with systematic review experience using published guidelines of the Cochrane Collaboration. The strategy was developed for PubMed/MEDLINE using keywords and MeSH terms (MEDLINE) (Supplementary File 1). To be as inclusive as possible, we limited the search strategy to terms covering NCDs and comorbidities among professional drivers and determinants or risk factors for NCDs in LMICs. We used keywords such as NCDs, comorbidities, hypertension, CVDs, diabetes and obesity, prevalence, risk factors, determinants, professional drivers, distant drivers, truck drivers, commercial drivers, LMICs and developing countries. This search strategy was also adapted to the other databases.

### Study selection

Study selection was managed using Covidence software (Australia). Two independent reviewers (B.J.N. and H.W.W.) evaluated articles for potential inclusion by screening titles and abstracts and assessed full publications to determine eligibility for final inclusion. Between each assessment, results were discussed to reach a consensus on the interpretation of inclusion criteria. Any further disagreements on study eligibility were resolved by consensus, and a third reviewer (H.P.N.) was consulted when necessary. Duplicate publications were identified and removed using the Covidence software version and manually. The PRISMA flow diagram showing the study selection process and reasons for exclusion is presented in Supplementary Figure 1.

### Data extraction

Two reviewers (B.J.N. and H.W.W.) independently extracted data using a standardized data extraction spreadsheet. The data were then compared, and any disagreements were resolved by consensus; the third reviewer (J.M.F.) was consulted when necessary. The data extraction process comprehensively captured information on the author, year of publication, country, study design, data collection period, sample size, response rate, participant and demographic and baseline characteristics, the types of NCDs and reported risk factors. We recorded the number of participants diagnosed with the selected NCDs and the total sampled drivers in the respective studies. Unavailable, unclear information and additional details were requested from the study investigators. Data were recorded in Excel spreadsheet 2020 (Microsoft Corporation, Redmond, WA).

### Quality assessment

Two reviewers (H.P.N. and J.M.F.) independently performed and rated the quality of the studies using the respective tools. We used and adapted the Joanna Briggs Institute (JBI) tool for cross-sectional prevalence studies (Munn *et al*., 2020) to assess the articles as low, moderate and high quality. Disagreement was resolved using arbitration by a third reviewer (B.J.N.). The tool encompassed nine questions with four responses: Yes, No, Unclear or Not applicable. A score of 1 was assigned for ‘Yes’ responses and 0 for all the other responses. The summary score for each article was obtained by summing the total number of ‘Yes’ responses. We then categorized the study quality score into low (0–3), medium (4–6) or high (7–9) quality.

### Data analysis

We analysed the data using Stata version 17.0. The weighted prevalence [95% confidence interval (CI)] of hypertension, DM, prediabetes, obesity and overweight were estimated using a random effect model and presented in forest plots. We anticipated high heterogeneity across studies due to differences in study populations, design and methods of outcome measurements. We, therefore, computed subgroup analysis by World Health Organization (WHO) regions (Africa, the Americas, the Eastern Mediterranean, Europe, South East Asia and the Western Pacific). We also stratified our findings based on the country’s income status, drivers’ driving distance and study period. Heterogeneity was assessed using *Q* and *I*^2^ statistics, where 75%, 50% and 25% indicated high, moderate and low heterogeneity, respectively (Higgins and Li, 2022). Publication bias was evaluated by visually examining the funnel plots of the effect size over standardized error. In addition, Egger’s linear regression test of funnel plot asymmetry was also performed (Egger *et al*., 1997). A *p-*value of less than 0.05 was considered statistically significant. All analyses were done in STATA 17 (StataCorp, 2023).

## RESULTS

### Article selection

Our article search obtained a total of 622 publications from six databases. After removing duplicates, 413 were eligible for title and abstract screening. Of 70 articles eligible for full-text screening, 41 met the inclusion criteria and were included in data extraction (Supplementary Figure 1).

### Characteristics of included studies and participants

Characteristics of the included studies are summarized in Table 1. Studies were from four continents: 20 were conducted in Asia and Mediterranean countries, 12 from Africa, 3 from Europe, and 6 from South America. The distributions by countries were as follows: India 10 (Joshi *et al*., 2013; Loukzadeh *et al*., 2013; Borle and Jadhao, 2015; Udayar *et al*., 2015; Jayakumar, 2017; Ravi *et al.*, 2017; Shahid and Chandra, 2017; Pushpa and Kanchana, 2018; Devi *et al*., 2021; Neralakatte *et al.*, 2021), Iran 7 (Saberi *et al.*, 2011; Rezaei Hachesu *et al.*, 2017; Montazerifar *et al*., 2019; Rezaei-hachesu, 2019; Shayestefar *et al*., 2019; Movahed *et al*., 2021), South Africa 5 (Adedokun *et al*., 2018, 2019; Lalla-Edward *et al*., 2019; Roche *et al*., 2021; Draaijer *et al*., 2022), Brazil 5 (Marqueze *et al.*, 2013; Smolarek *et al*., 2013; Sangaleti *et al.*, 2014; Reis *et al*., 2017; Souza *et al.*, 2019), Nigeria 4 (Amadi *et al*., 2018; Ogbonnaya *et al*., 2019; Showande and Odukoya, 2020; Ibitoba *et al*., 2022), Ghana 2 (Anto *et al*., 2020; Appiah *et al.*, 2020), Turkey 2 (Hayran *et al.,* 2009; Özdemir *et al*., 2009), Thailand 1 (Kaewboonchoo *et al.*, 2007), Russia 1 (Zhidkova *et al*., 2022), Peru 1 (Quichua *et al*., 2021), China 1 (Siu *et al*., 2012), Egypt 1 (Mohsen and Hakim, 2019) and Ethiopia 1 (Yosef *et al*., 2020). All studies were designed cross-sectionally and published between 2007 and 2022. A total of 48 414 study participants were studied; the age range was 18–77 years. Most studies reported hypertension, DM and obesity among male drivers; only five studies had a small proportion of female drivers included in the study, with a proportion ranging from 1.2% to 12.6%, and most participants were bus drivers. In the 10 studies with data on work duration, the mean work duration ranged from 5 to 25 years (Table 1).

**Table 1:** Characteristics of the included studies

Author (year)	Country	WHO region	Study design	Study period	Study population	Sample	Mean age	Age range	Mean work duration (years)	Female (%)	Quality score
Adedokun *et al*. (2018)	South Africa	Africa	Cross-sectional	2017	Commercial drivers	403	43.3 ± 12.5	20–74		1.2	8
Adedokun *et al.* (2019)	South Africa	Africa	Cross-sectional	2017	Commercial drivers	403	43.3 ± 12.5			1.2	8
Amadi *et al.* (2018)	Nigeria	Africa	Cross-sectional	2015	Long-distance bus drivers	308	44.8 ± 9.7	25–76		0	8
Anto *et al*. (2020)	Ghana	Africa	Cross-sectional	2015–2016	Metro mass bus drivers	527	44.1 ± 9.3				6
Appiah *et al*. (2020)	Ghana	Africa	Cross-sectional	2019	Motor vehicle drivers	100	41 ± 8.9	25–69		0	2
Borle and Jadhao (2015)	India	Southeast Asia	Cross-sectional	2010–2011	Occupational bus drivers	587	46.9 ± 6.7	28–57	24.47 ± 7.4	0	6
Devi *et al*. (2021)	India	Southeast Asia	Cross-sectional	2019	Auto rickshaw drivers	159		20–65			4
Draaijer *et al.* (2022)	South Africa	Africa	Cross-sectional	2016–2017	long-distance truck driver	614	37 (31–42)		9 (5–14)	0	9
Rezaei Hachesu *et al*. (2017)	Iran	Eastern Mediterranean	Cross-sectional	2016	Taxi drivers	110	46.5(11.4)		11.36(8.85)	0	5
Rezaei-hachesu (2019)	Iran	Eastern Mediterranean	Cross-sectional	2016	Taxi drivers	120	46.7 ± 12.0		11.31 ± 8.07	0	5
Hayran *et al*. (2009)	Turkey	Europe	Cross-sectional	2004	Bus drivers	5040				0	9
Ibitoba *et al.* (2022)	Nigeria	Africa	Cross-sectional	2021	Commercial motorcyclists	310				0	9
Shahid and Chandra (2017)	India	Southeast Asia	Cross-sectional	2015–2016	Drivers	400		26–57		0	8
Jayakumar (2017)	India	Southeast Asia	Cross-sectional	2008	Railway loco pilots (engine drivers)	230				0	5
Joshi *et al.* (2013)	India	Southeast Asia	Cross-sectional	2012	Bus drivers	400				0	8
Kaewboonchoo *et al.* (2007)	Thailand	Southeast Asia	Cross-sectional	2002	Bus drivers	444	42 ± 8	23–59	10 ± 7	0	4
Lalla-Edward *et al.* (2019)	South Africa	Africa	Cross-sectional	2016–2017	Truck drivers	614	37 (31–42)		9 (5–14)	0	8
Loukzadeh *et al.* (2013)	India	Southeast Asia	Cross-sectional	2012	Train drivers	152	36 ± 8.8		8 (8)		6
Marqueze *et al.* (2013)	Brazil	Americas	Cross-sectional	2009	Truck drivers	57	39.8 ± 6.6	29–56	15.7	0	6
Mohebbi *et al.* (2012)	Iran	Eastern Mediterranean	Cross-sectional	2007–2010	Professional drivers	12 138	37.8 ± 10.1	20–67		0	9
Mohsen and Hakim (2019)	Egypt	Eastern Mediterranean	Cross-sectional	2016–2017	Bus drivers	234	37.4 ± 9.0			0	7
Montazerifar *et al.* (2019)	Iran	Eastern Mediterranean	Cross-sectional	2015–2016	Professional drivers (bus and taxi)	500				0	6
Movahed *et al.* (2021)	Iran	Eastern Mediterranean	Cross-sectional	2017–2018	Commercial drivers	903	42.8 ± 10.1			0	4
Neralakatte *et al.* (2021)	India	Southeast Asia	Cross-sectional	2020	Auto drivers	450	40.6 ± 8.9				6
Ogbonnaya *et al.* (2019)	Nigeria	Africa	Cross-sectional	2018	Commercial drivers	103	43.2 ± 12.3	24–75		0	8
Özdemir *et al*. (2009)	Turkey	Europe	Cross-sectional	2004–2006	Bus and truck drivers	200	40.3 ± 8.6			0	8
Pushpa and Kanchana (2018)	India	Southeast Asia	Cross-sectional	2017	Bus drivers	30	31.4 ± 4.1		5.15 ± 2.84	0	6
Quichua *et al.* (2021)	Lima/Peru	Americas	Cross-sectional	2019	Bus drivers	195	39 (47–32)	18–70		6.3	9
Ravi *et al*. (2017)	India	Southeast Asia	Cross-sectional	2018	Truck drivers	3200	40.3 ± 11.4				8
Reis *et al*. (2017)	Brazil	Americas	Cross-sectional	2014	Truck drivers	155	41 (21–72)			0	5
Roche *et al.* (2021)	South Africa	Africa	Cross-sectional	2020	Truck drivers	614	37.7 ± 9.0		10.0 ± 7.0	0	7
Saberi *et al.* (2011)	Iran	Eastern Mediterranean	Cross-sectional	2007	Bus and truck drivers	429	36.6 ± 10.7	21–73		0	4
Sangaleti *et al.* (2014)	Brazil	Americas	Cross-sectional	2010–2011	Truck drivers	250	41.9 ± 10	22–60		0	8
Shayestefar *et al.* (2019)	Iran	Eastern Mediterranean	Cross-sectional	2018	Drivers	948	44.2 ± 11.7	22–69		0	7
Showande and Odukoya (2020)	Nigeria	Africa	Cross-sectional	2014	Commercial drivers	152		20–77		0	8
Siu *et al.* (2012)	China	Western Pacific	Cross-sectional	2007–2010	Professional drivers	3482	50.9 (7.6)			7.4	6
Smolarek *et al.* (2013)	Brazil	Americas	Cross-sectional	2012	Bus drivers	75	38.6 ± 5.7			0	2
Souza *et al.* (2019)	Brazil	Americas	Cross-sectional	2012	Urban transportation workers	1126				12.6	9
Udayar *et al*. (2015)	India	Southeast Asia	Cross-sectional	2012	Transport drivers	244	41.4 ± 10.4			0	3
Yosef *et al.* (2020)	Ethiopia	Africa	Cross-sectional	2018	Long-distance truck drivers	422	37.7 ± 9.13	22–59			9
Zhidkova *et al.* (2022)	Russia	Europe	Cross-sectional	2021	Railway drivers and assistants	11 059	38.3 ± 10.2				4

The included studies applied different definitions of hypertension; the majority defined hypertension according to the WHO guideline for hypertension management and the local national-level guidelines as a systolic blood pressure of 140 mmHg or diastolic blood pressure of 90 mmHg and above or both (WHO, 2021). Thirteen studies used fasting blood glucose (FBG) for the diagnosis of DM, which is defined as FBG of ≥7.0 mmol/l (≥126 mg/dl), and prediabetes as FBG of 5.6–6.9 mmol/l (100–125 mg/dl) (WHO/UCN/NCD/20.1, 2020). The remaining 13 studies reported the diagnosis of DM based on random blood glucose (RBG), defined as RBG of >11.1 mmol/l (>200 mg/dl). All except two studies used body mass index to diagnose obesity and overweight (Supplementary Table 2).

### Study quality assessment

The quality assessment showed that about half of the studies (52%, *n* = 21) were of high quality. Seventeen studies (41%) were of moderate quality, and three (7%) were of low quality (Smolarek *et al*., 2013; Udayar *et al*., 2015; Appiah *et al*., 2020). Poor quality recorded for the three studies was mainly due to small sample sizes, unclear outcome assessment methods and the use of non-probabilistic sampling methods (Supplementary Table 1).

### Prevalence of hypertension

**Fig. 1:**
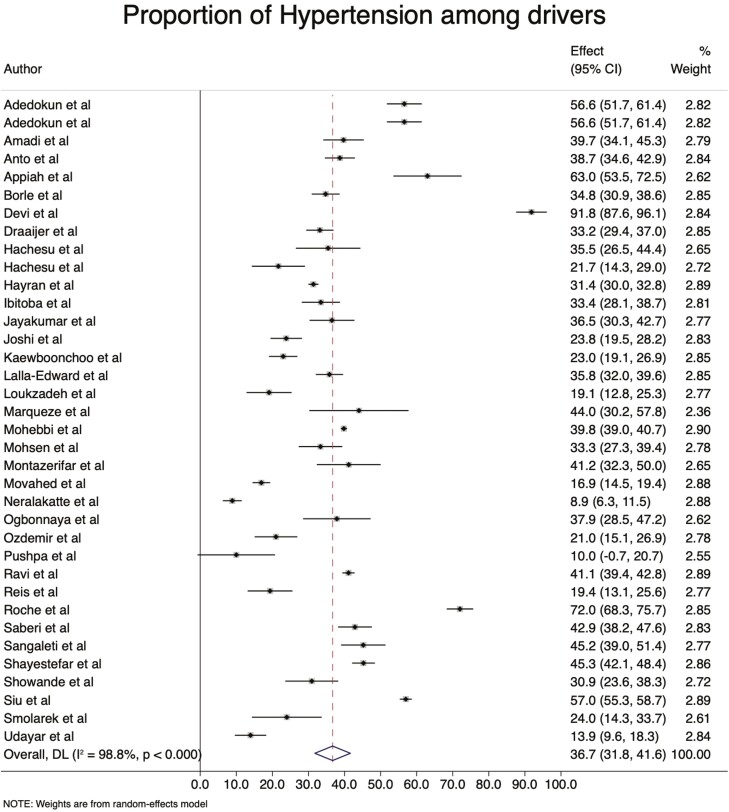
The prevalence of hypertension among professional drivers.

Out of 41 included studies, 36 (87.8%) were included in the meta-analysis to assess hypertension. The weighted prevalence of hypertension among professional drivers was 36.7% (95% CI: 31.8–41.6%). The highest estimated prevalence was reported at 91.8% (Devi *et al*., 2021) and the lowest at 8.9% (Neralakatte *et al*., 2021), both from India (Figure 1).

### Prevalence of DM and prediabetes

**Fig. 2:**
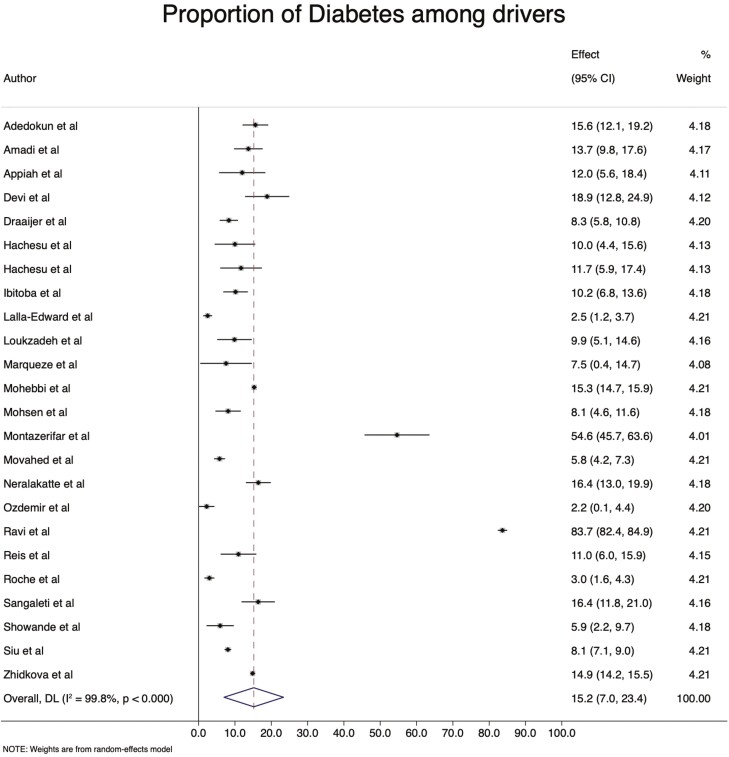
The prevalence of DM among professional drivers.

**Fig. 3:**
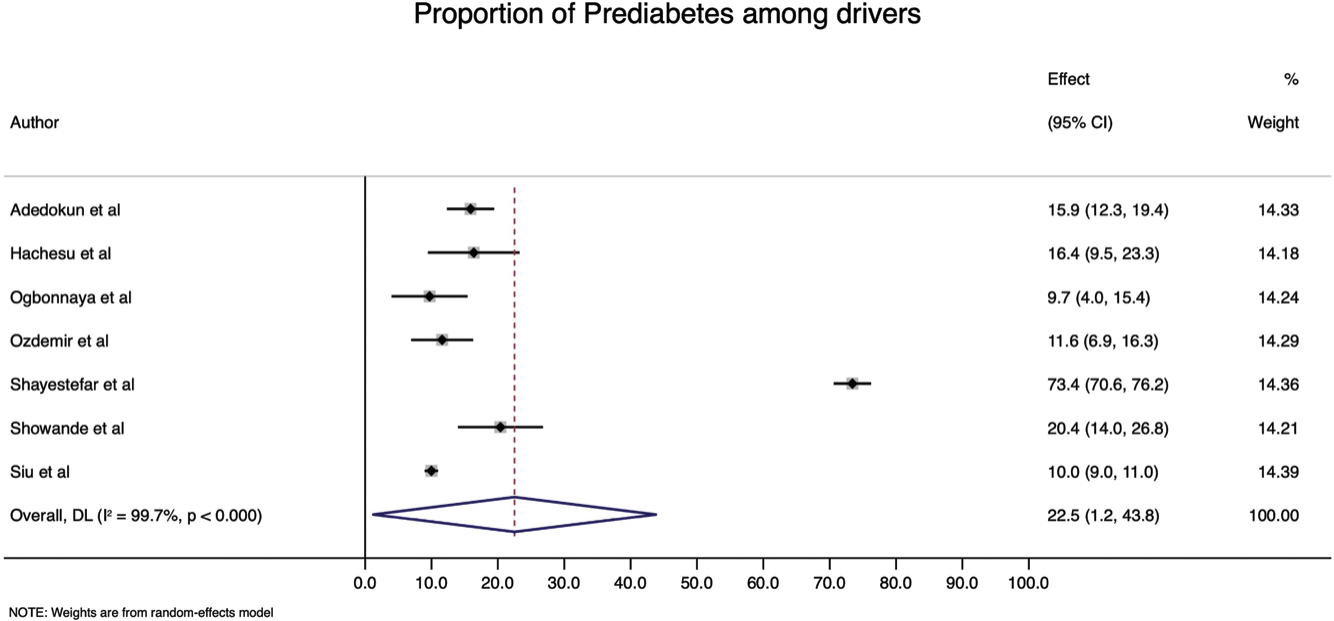
The prevalence of prediabetes among professional drivers.

Twenty-six (63.4%) studies were included in the meta-analysis to determine the pooled DM estimate. The pooled prevalence of DM is 15.2% (95% CI: 7.0–23.4%), ranging from 2.2% among drivers in Turkey (Özdemir *et al*., 2009) to 83.7% in India (Ravi *et al*., 2017) (Figure 2). The prevalence of prediabetes was 22.5% (95% CI: 1.2–43.8%), as reported by seven (17.1%) included studies (Figure 3).

### Prevalence of obesity and overweight

**Fig. 4:**
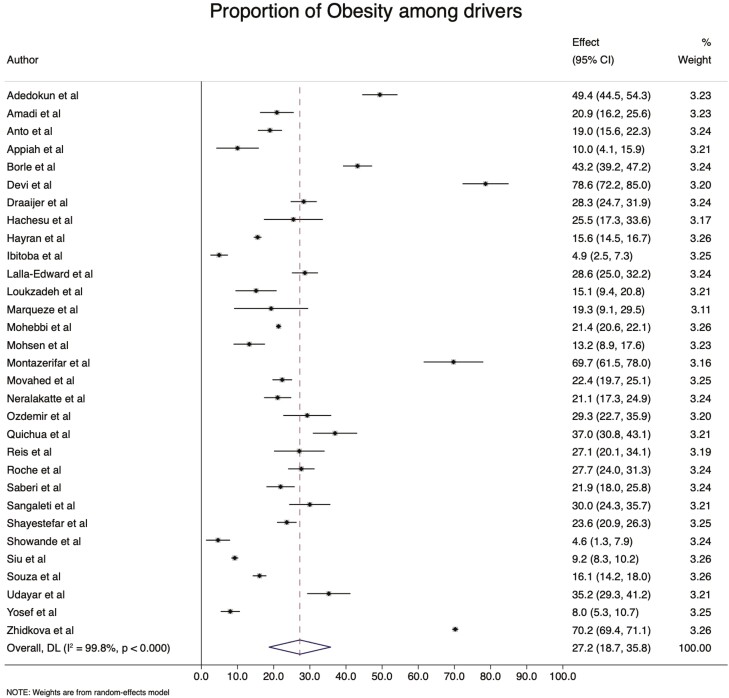
The prevalence of obesity among professional drivers.

**Fig. 5:**
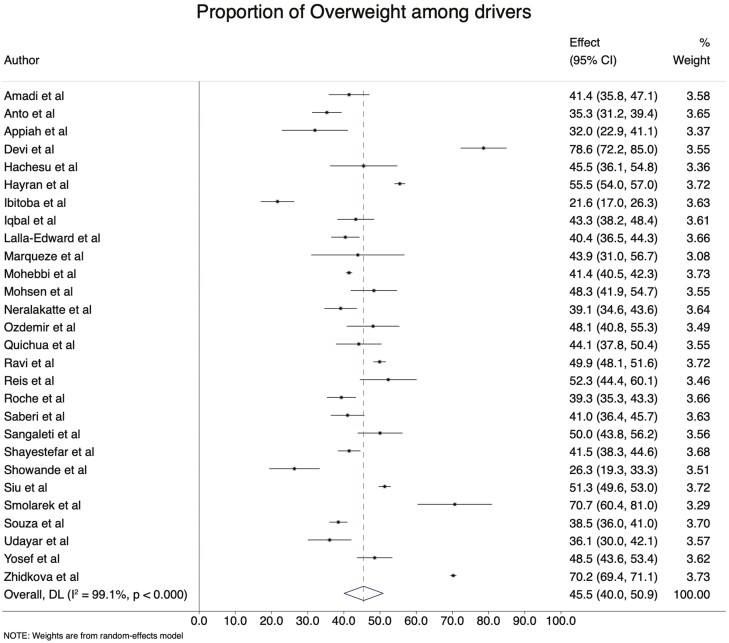
The prevalence of overweight among professional drivers.

Thirty-four (81.0%) studies were included to assess the pooled obesity estimate. The prevalence of obesity ranged from 4.6% in Nigeria (Showande and Odukoya, 2020) to 78.6% in India (Devi *et al*., 2021). The pooled prevalence of obesity and overweight is 27.0 (95% CI: 18.7–35.3%) (Figure 4) and 45.1 (95% CI: 39.8–50.4%) (Figure 5), respectively.

### Publication bias

We evaluated the possibility of publication bias using visual inspection (funnel plot) and an objective measure (Egger’s test). The funnel plot showed an equal distribution of studies from the line of effect. The results from Egger’s test showed no significant small study effect bias for hypertension (*p* = 0.642), diabetes (*p* = 0.911), prediabetes (*p* = 0.500), obesity (*p* = 0.604) and overweight estimates (*p* = 0.073).

### Subgroup analyses of the prevalence of hypertension, DM and obesity by WHO regions, country income status, driving distance and study period

The pooled prevalence of hypertension, DM and obesity was further analysed by the WHO region. The prevalence of hypertension differed by WHO region (*p* = 0.02); the highest was reported in the Western Pacific (57.0%), followed by Africa (45.2%), and the lowest was in Europe (27.0%). There was no significant difference in the prevalence of DM by WHO regions (*p* = 0.12); however, the highest prevalence of DM was reported in Southeast Asia (32.3%) (Figure 5). Southeast Asia (38.6%) and Europe (38.4%) had the highest prevalence of obesity, while the West Pacific had the lowest (9.2%) (*p* < 0.01) (Supplementary Figures 2, 3, 4).

There was no significant difference in the prevalence of hypertension (*p* = 0.37), DM (*p* = 0.07) and obesity (*p* = 0.47) by country income status. Professional drivers engaging in long-distance driving did not have a significantly higher prevalence of hypertension (*p* = 0.28), DM (*p* = 0.57) or obesity (*p* = 0.47) compared to those not engaging in long-distance driving. The highest prevalence of hypertension was reported between 2007 and 2011 (43.0%) and from 2017 to 2021 (42.4%) (*p* < 0.01). However, the prevalence of DM (*p* < 0.01) has been significantly increasing (Supplementary Figures 2, 3, 4).

### Factors associated with hypertension, DM and obesity among professional drivers

Three studies reported age as a significant associated factor for hypertension in professional drivers (Hayran *et al*., 2009; Adedokun *et al*., 2018; Amadi *et al*., 2018). The odds of hypertension increased with increasing age; Adedokun and colleagues reported over two times the likelihood of hypertension for drivers aged 35 years and above (Adedokun *et al*., 2018). Overweight and obesity increased the odds of hypertension in four studies (Hayran *et al*., 2009; Reis *et al*., 2017; Adedokun *et al*., 2018; Amadi *et al*., 2018); obese drivers in Turkey were 4.6 times more likely to be hypertensive compared to their counterparts (Hayran *et al*., 2009). Other associated factors were DM, work duration, alcohol use (Adedokun *et al*., 2018), smoking, high cholesterol levels (Hayran *et al*., 2009), increased abdominal circumference and family history of CVD (Sangaleti *et al*., 2014). Participants who were old (Siu *et al*., 2012; Adedokun *et al*., 2019), married (Adedokun *et al*., 2019), obese (Sangaleti *et al*., 2014; Amadi *et al*., 2018), hypertensive (Adedokun *et al*., 2019) and with a positive family history of DM (Siu *et al*., 2012) were reported to be more likely to have DM. Risk factors for obesity and overweight included physical inactivity (Reis *et al*., 2017; Anto *et al*., 2020), sitting for long durations (Anto *et al*., 2020; Yosef *et al*., 2020), eating late at night (Anto *et al*., 2020), using sleep inhibitors, high-calorie intake, eating under stressful conditions (Anto *et al*., 2020), monthly income of >220 USD, family sizes of three more members and sleeping less than 3 hours (Yosef *et al*., 2020) (Supplementary Table 2).

### Recommended interventions from the included studies

Different studies reported recommendations for tackling the high burden of NCDs among professional drivers. We summarized the recommendations in four main themes: (i) health promotion through educational programs and public awareness of lifestyle modifications; (ii) regular screening services for NCDs and associated risk factors; (iii) improvement of policies governing the transportation sectors such as driving hours and organization of work and (iv) establishing facilities for physical exercise and recreation (Supplementary Table 3).

## DISCUSSION

This review aimed to collate evidence on the burden of hypertension, DM and obesity and reported diverse and modifiable factors increasing the risk of NCDs among professional drivers. Our review reported findings from 41 studies, including 48 414 study participants, that reported the prevalence of the three common NCDs (hypertension, diabetes and obesity) among professional drivers. The pooled prevalence of hypertension, diabetes and obesity was 36.7%, 15.2% and 27.0% respectively. Additionally, 45.1% of all included participants were overweight, and 22.5% had prediabetes. Included studies reported age, overweight, obesity, diabetes, work duration, alcohol use, smoking, high cholesterol levels and family history of CVD as risk factors for hypertension. Participants at risk of DM were older, married, obese, hypertensive and with a positive family history of DM. Physical inactivity and sedentary lifestyle, poor eating practices, using sleep inhibitors and sleeping less than 3 hours were associated with increased odds of obesity.

The hypertension prevalence among professional drivers reported in this study (36.7%) is slightly higher than the overall prevalence of hypertension reported globally (31.1%) in the year 2020 (Mills *et al*., 2020). Given the projected 30% increase in hypertension prevalence in the general population by the year 2025 (Kearney *et al*., 2005), our findings imply that the prevalence of hypertension among professional drivers will be approaching an alarming proportion (42%). With a growing body of evidence indicating a higher projected increase in hypertension prevalence in LMICs compared to high-income countries (HICs), it is reasonable to assume that the prevalence gap between professional drivers and the general population will be more pronounced in LMICs.

In 2021, the age-standardized global DM prevalence was 6.1% in the general population; three-quarters of the DM burden was in LMICs (Ong *et al*., 2023). Similar evidence reported twice the burden in low-income countries compared to HICs (Saeedi *et al*., 2019). In our context, the overall prevalence of 15.2% among professional drivers in LMICs is more than twice that of the general population. This further substantiates that professional drivers, due to the nature of their occupation that puts them at a higher risk for NCDs, are more affected than the general population (Gona *et al*., 2021). West Pacific (China) and the Americas regions had the highest DM burden in the current study, similar to the 2021 Global Burden of Disease that showed higher DM prevalence in the Middle East and Latin America regions (Ong *et al*., 2023).

Sedentary behaviours significantly contribute to overweight and obesity (Silveira *et al*., 2022). Given that professional driving is a predominantly sedentary job, our study findings further support the association between sedentary occupations and the increased risk of overweight and obesity. In 2019, 8.8% of adult males and 18.5% of adult females were reported to be obese in LMICs (Gona *et al*., 2021). Our study found that 27.0% of all professional drivers were obese, a prevalence almost 3.07-fold higher than the 8.8% reported among adult males in the general population.

On average, humans spend more than half of their lives engaging in different employment (International Labour Office, 2012). Professional drivers represent a key occupational category that is predominantly sedentary during their working hours. The impact is significantly higher for long-distance driving compared to short distances, with a 4-fold increase in the likelihood of developing NCDs for long-distance drivers (Yosef *et al*., 2020). Other behavioural patterns that predispose them to NCD risk factors include poor eating behaviours and interrupted sleep patterns with day-night shifts that affect their circadian rhythm (Olson *et al*., 2017). All of these are known to be the main contributors to NCD in adults (Gómez-Olivé *et al*., 2018; Twinamasiko *et al*., 2018).

Additionally, professional drivers are not spared from the other known risk factors for hypertension, diabetes and obesity, such as excessive alcohol consumption and tobacco use, as reported elsewhere (Mills *et al*., 2020; Ong *et al*., 2023). Moreover, some of the reported complications of NCDs, such as diabetic retinopathy, neuropathy or medication-induced hypoglycaemia, can impact driving skills and practice (Tamilarasan *et al*., 2023). Further, there are no established screening interventions for individuals in the driving occupation; it is not only difficult to detect NCDs early for this population, but it is also challenging for the drivers to adhere to treatment under these occupation conditions, leading to adverse health outcomes at the individual level as well as increased risk of road traffic accidents (Almigbal *et al*., 2018).

### Limitations

Our study should be interpreted in the light of the following limitations. A few studies in our analyses were of low quality due to small sample sizes and the use of non-probabilistic sampling methods, which could have decreased the studies’ power. The high heterogeneity in our pooled estimates of the included studies was another limitation; this may be due to differences in the study contexts, sampled populations and outcome measurements. On diagnosis-related limitations, most studies did not document three blood pressure readings as recommended in hypertension diagnosis, and 50% of studies used RBG alone in documenting DM. Only a few studies reported on the risk factors for NCDs among drivers, so our ability to estimate the pooled risk factors for NCDs among drivers was limited. Other key factors, such as dietary habits and patterns, physical activity, smoking and alcohol intake, were also not documented in the included studies.

## CONCLUSION AND RECOMMENDATION

To the best of our knowledge, this is the first systematic review study to report the burden of NCDs among one of the predominantly sedentary occupations, professional drivers, in LMICs. The prevalence of the common NCDs among professional drivers is higher than the reported burden in the general population. The prevalence of DM and obesity were 2- and 3-fold higher compared to findings in general populations, respectively. In addition to the general NCD risk factors, long sitting hours, unhealthy eating behaviours and inconsistent sleep patterns commonly affect professional drivers, predisposing them to a higher likelihood of acquiring NCDs.

Our findings highlight the alarming burden of NCDs on professional drivers whose risks are linked to their occupations. The WHO recommends designing workplaces as healthy settings to promote health and well-being, including prevention, control and interventions for NCDs; this is also highlighted in the WHO Global Action Plan for Worker’s Health (Fletcher and Crawford, 2008). This aligns with the global target to reduce physical inactivity in adults and adolescents by 15% by 2023 (WHO, 2018) and the recommended best buys for the prevention and control of NCDs (WHO, 2024). Incorporating NCDs-related interventions at workplaces for professional drivers will provide the most convenient opportunity for detecting and controlling NCDs in this predominantly sedentary population. Such interventions must be tailored to include both preventive and treatment measures for NCDs. We recommend such interventions as useful in combating different occupation-related risk factors for NCDs, such as unsupportive environment, work stress, sedentary lifestyle, consumption of unhealthy foods and shift work (Pg Ismail and Koh, 2014). We recommend interventions such as facilities for physical exercises, psychological support at work, dissemination of educational content on dietary intake and lifestyle, substance use cessation support, education and regulations, and regular NCD screening and referral services at workplaces (International Labour Office, 2012). These health promotion, prevention and intervention strategies for NCDs among professional drivers will require a multisectoral approach beyond the healthcare system (Alwan and MacLean, 2009) and are urgently needed, given the high prevalence reported in this review.

## Supplementary Material

daae087_suppl_Supplementary_Files_1

daae087_suppl_Supplementary_Figures_1

daae087_suppl_Supplementary_Tables_2

daae087_suppl_Supplementary_Tables_1

daae087_suppl_Supplementary_Figures_2

daae087_suppl_Supplementary_Figures_3

daae087_suppl_Supplementary_Figures_4

daae087_suppl_Supplementary_Tables_3

## Data Availability

The data underlying this article will be shared on reasonable request to the corresponding author.
