# Correction to: Epidemiology of non-communicable diseases among professional drivers in LMICs: a systematic review and meta-analysis

**DOI:** 10.1093/heapro/daae125

**Published:** 2024-09-16

This is a correction to: Belinda J Njiro, Harrieth P Ndumwa, Hannah Wanjiku Waithera, Rehema Chande, William Julius, Fredirick Mashili, Julius C Mwita, Monica H Swahn, Catherine Staton, Joel Msafiri Francis, Epidemiology of non-communicable diseases among professional drivers in LMICs: a systematic review and meta-analysis, *Health Promotion International*, Volume 39, Issue 4, August 2024, daae087, https://doi.org/10.1093/heapro/daae087.

In the originally published PDF version of this manuscript, there was a typographic error in the title. This has been corrected. The publisher apologizes for the error.

