# Supplementary material for: Epidemiology of non-communicable diseases among professional drivers in LMICs: a systematic review and meta-analysis
Source: Health Promot Int. 2024 Aug 31;39(4):daae087. doi: 10.1093/heapro/daae087 (PMC11364521; doi:10.1093/heapro/daae087)
Supplement: daae087_suppl_Supplementary_Files_1 [file daae087_suppl_supplementary_files_1.docx]

**Supplementary File 1: Search strategies**

1. **PubMed**

| **Search** | **Query** |
| --- | --- |
| #1 **Noncommunicable Diseases** | "Noncommunicable Diseases"[Mesh] OR "Noncommunicable Disease*"[tiab] OR "Non-infectious Disease*"[tiab] OR "Non infectious Disease*"[tiab] OR "Non-communicable Disease*"[tiab] OR "Non communicable Disease*"[tiab] OR "Noninfectious Disease*"[tiab] OR comorbidit*[tiab] OR hypertension[tiab] OR "cardiovascular disease*"[tiab] OR diabet* OR obesity[tiab] |
| #2 - **Prevalence** | "Prevalence"[Mesh] OR "epidemiology" [Subheading] OR "Risk Factors"[Mesh] OR "Cardiometabolic Risk Factors"[Mesh] OR "Heart Disease Risk Factors"[Mesh] OR "Epidemiologic Factors"[Mesh] OR "Social Determinant*"[tiab] OR "Risk factor*"[tiab] OR prevalence*[tiab] OR determinant*[tiab] |
| #3 – **Driver** | "Automobile Driving"[Mesh] OR distant driver*[tiab] OR "truck driver*"[tiab] OR "commercial driver*"[tiab] OR driver*[tiab] |
| #4 **Developing countries** | "Developing Countries"[Mesh] OR "middle-income countr*"[tiab] OR LMICs[tiab] OR "Low and Middle Income Countr*"[tiab] OR "Lower Middle Income Countr*"[tiab] OR "Less Developed Countr*"[tiab] OR "Less-Developed Countr*"[tiab] OR "Third World Countr*"[tiab] OR "Third-World Countr*"[tiab] OR "Third-World Nation*"[tiab] OR "Developing Nation*"[tiab] OR "Least Developed Countr*"[tiab] OR "Low Income Countr*"[tiab] OR "Third-World Countr*"[tiab] |
| **#5** | **#1 AND #2 AND #3 AND #4** |

Search done on 11^th^ May 2023

1. **EMBASE Top of Form**

| **Search** | **Query** |
| --- | --- |
| #1 **Noncommunicable Diseases** | 'noncommunicable disease*' OR 'non-infectious disease*' OR 'non infectious disease*' OR 'non-communicable disease*' OR 'noninfectious disease*' OR comorbidit* OR 'hypertension'/exp OR hypertension OR 'cardiovascular disease*' OR diabet* OR 'obesity'/exp OR obesity |
| #2 – **Epidemiology/ Prevalence** | 'prevalence*' OR 'epidemiology' OR 'risk factor*' OR 'cardiometabolic risk factor*' OR 'heart disease risk factor*' OR 'epidemiologic factor*' OR 'social determinant*' OR determinant* |
| #3 – **Professional Drivers** | 'automobile driving' OR 'truck driver*' OR 'distant driver*' OR 'commercial driver*' OR driver* |
| #4 **Developing countries** | 'developing countr*' 'middle-income countr*' lmics 'low and middle income countr*'  'lower middle income countr*' 'less developed countr*' 'less-developed countr*' 'third  world countr*' 'third-world nation*' 'developing nation*' 'least developed countr*' 'low  income countr*' |
| **#5** | **#1 AND #2 AND #3 AND #4** |

Bottom of Form

Bottom of Form

Search done on 19^th^ April 2023

1. **SCOPUS Top of Form**

| **Search** | **Query** |
| --- | --- |
| #1 **Noncommunicable Diseases** | TITLE-ABS-KEY (“noncommunicable disease*” OR “non-infectious disease*” OR ''non-communicable disease*” OR “non infectious disease*” OR comorbidit* OR hypertension* OR “cardiovascular disease*” OR diabet* OR obesit*) |
| #2 – **Epidemiology/ Prevalence** | TITLE-ABS-KEY ( "prevalence*" OR "epidemiology" OR 'risk factor*' OR "cardio metabolic risk factor*" OR "heart disease risk factor" OR "epidemiologic factor*" OR "social determinant*" OR determinant* ) |
| #3 – **Professional Drivers** | TITLE-ABS-KEY ('automobile driving' OR 'truck driver*' OR 'distant driver*' OR 'commercial driver*' OR driver*) |
| #4 **Developing countries** | TITLE-ABS-KEY ( "developing countr*" OR "middle-income countr*" OR lmics OR "low and middle income countr*" OR "lower middle income countr*" OR "less developed countr*" OR "less-developed countr*" OR "third world countr*" OR "third-world nation*" OR "developing nation*" OR "least developed countr*" OR "low income countr*") |
| **#5** | **#1 AND #2 AND #3 AND #4** |

Search done on 11/05/2023

1. **Web of Science**

| **Search** | **Query** |
| --- | --- |
| #1 **Noncommunicable Diseases** | ("Noncommunicable Disease*" ) OR ("Non-infectious Disease*") OR ("Non infectious Disease*") OR ("Non-communicable Disease*") OR ("Noninfectious Disease*") OR (comorbidit*) OR ( hypertension) OR ( "cardiovascular disease*") OR ( diabet* ) OR (obesity) |
| #2 – **Prevalence** | ("Prevalence*" ) OR ("epidemiology") OR ("Risk Factor*") OR ("Cardiometabolic Risk Factor*") OR ("Heart Disease Risk Factor*") OR ("Epidemiologic Factor*") OR ("Social Determinant*") OR (determinant*) |
| #3 – **Professional Driver** | ("Automobile Driving") OR ("truck driver*") OR ("distant driver*") OR ( "commercial driver*") OR (driver*) |
| #4 **Developing countries (Low and Middle Income Countries)** | ("Developing Countr*") OR ("middle-income countr*") OR (LMICs) OR ("Low and Middle Income Countr*") OR ("Lower Middle Income Countr*" ) OR ("Less Developed Countr*") OR ("Less-Developed Countr*") OR ("Third World Countr*") OR ("Third-World Nation*") |
| **#5** | **#1 AND #2 AND #3 AND #4** |

Search done on 10^th^ May, 2023

1. **Global Health**

| **Search** | **Query** |
| --- | --- |
| #1 **Noncommunicable Diseases** | ("Noncommunicable Disease*") OR ("Noncommunicable Disease*") OR ("Non-infectious Disease*") OR ("Non infectious Disease*") OR ("Non-communicable Disease*") OR ("Noninfectious Disease*") OR (comorbidit*) OR ( hypertension) OR ( "cardiovascular disease*") OR ( diabet* ) OR (obesity) |
| #2 – **Prevalence** | ("Prevalence*") OR ("epidemiology") OR ("Risk Factor*") OR ("Cardiometabolic Risk Factor*") OR ("Heart Disease Risk Factor*") OR ("Epidemiologic Factor*") OR ("Social Determinant*") OR (determinant*) |
| #3 – **Professional Driver** | ("Automobile Driving") OR ("truck driver*") OR ("distant driver*") OR ( "commercial driver*") OR (driver*) |
| #4 **Developing countries (Low and Middle Income Countries)** | ("Developing Countr*") OR ("middle-income countr*") OR (LMICs) OR ("Low and Middle Income Countr*") OR ("Lower Middle Income Countr*") OR ("Less Developed Countr*") OR ("Less-Developed Countr*") OR ("Third World Countr*") OR ("Third-World Nation*") |
| **#5** | **#1 AND #2 AND #3 AND #4** |

Search conducted on 10^th^ May, 2023

1. **Africa Wide Information (AWI)**

| **Search** | **Query** |
| --- | --- |
| #1 **Noncommunicable Diseases** | ("Noncommunicable Disease*" ) OR ("Non-infectious Disease*") OR ("Non infectious Disease*") OR ("Non-communicable Disease*") OR ("Noninfectious Disease*") OR (comorbidit*) OR ( hypertension) OR ( "cardiovascular disease*") OR ( diabet* ) OR (obesity) |
| #2 – **Prevalence** | ("Prevalence*" ) OR ("epidemiology") OR ("Risk Factor*") OR ("Cardiometabolic Risk Factor*") OR ("Heart Disease Risk Factor*") OR ("Epidemiologic Factor*") OR ("Social Determinant*") OR (determinant*) |
| #3 – **Professional Driver** | ("Automobile Driving") OR ("truck driver*") OR ("distant driver*") OR ( "commercial driver*") OR (driver*) |
| #4 **Developing countries (Low and Middle Income Countries)** | ("Developing Countr*") OR ("middle-income countr*") OR (LMICs) OR ("Low and Middle Income Countr*") OR ("Lower Middle Income Countr*" ) OR ("Less Developed Countr*") OR ("Less-Developed Countr*") OR ("Third World Countr*") OR ("Third-World Nation*") |
| **#5** | **#1 AND #2 AND #3 AND #4** |

Search conducted on 10^th^ May, 2023
