# Supplementary figures and images for: Epidemiology of non-communicable diseases among professional drivers in LMICs: a systematic review and meta-analysis
Source: Health Promot Int. 2024 Aug 31;39(4):daae087. doi: 10.1093/heapro/daae087 (PMC11364521; doi:10.1093/heapro/daae087)

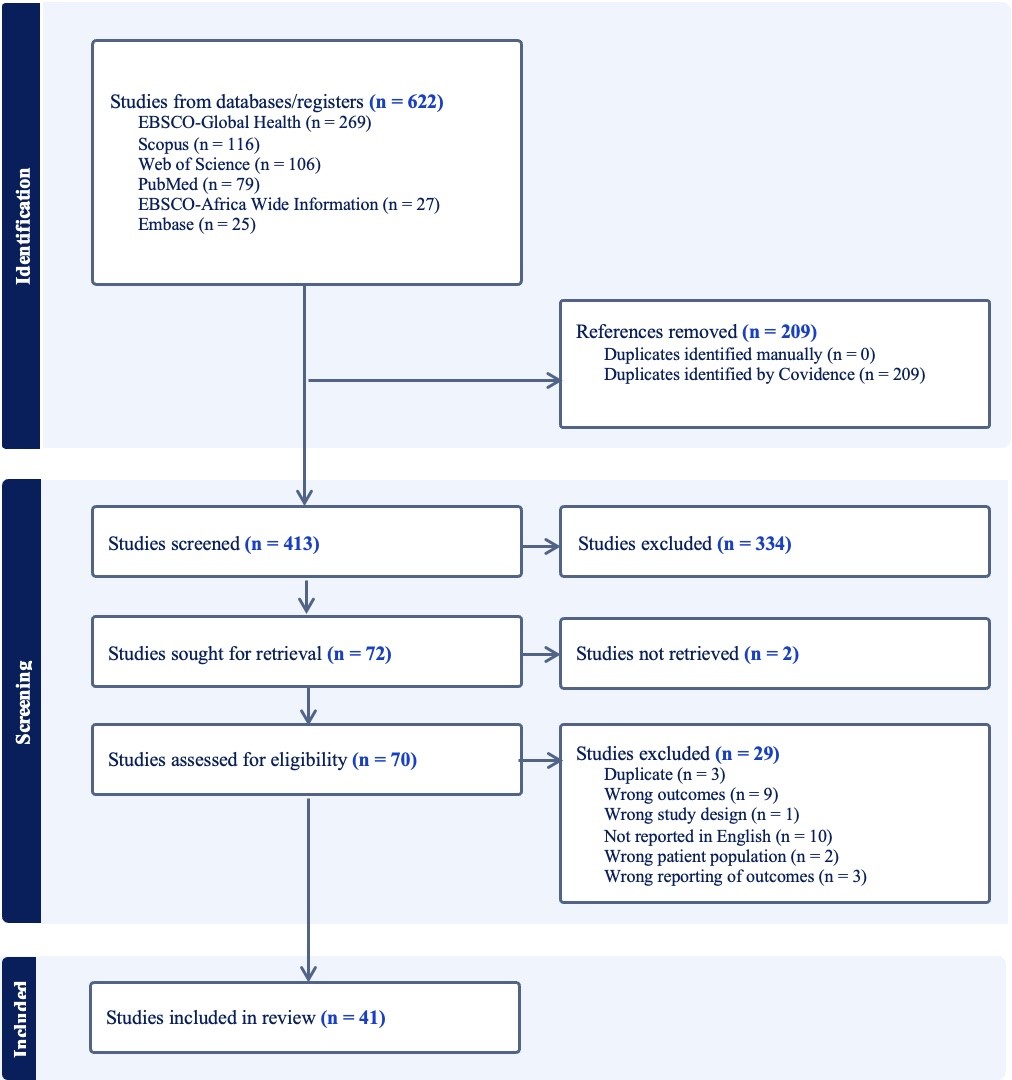

Supplement: daae087_suppl_Supplementary_Figures_1 [file daae087_suppl_supplementary_figures_1.jpeg]

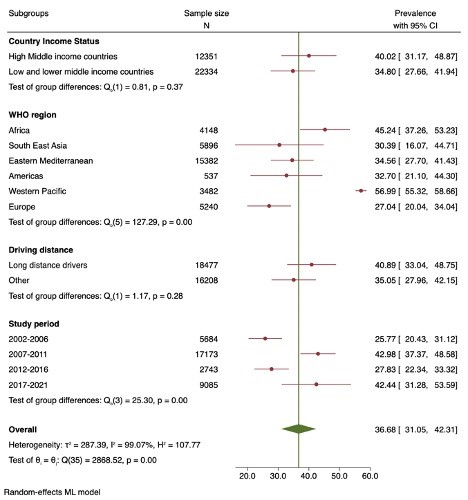

Supplement: daae087_suppl_Supplementary_Figures_2 [file daae087_suppl_supplementary_figures_2.jpeg]

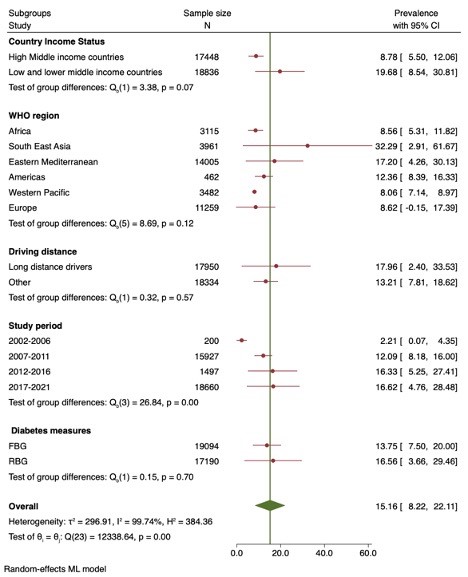

Supplement: daae087_suppl_Supplementary_Figures_3 [file daae087_suppl_supplementary_figures_3.jpeg]

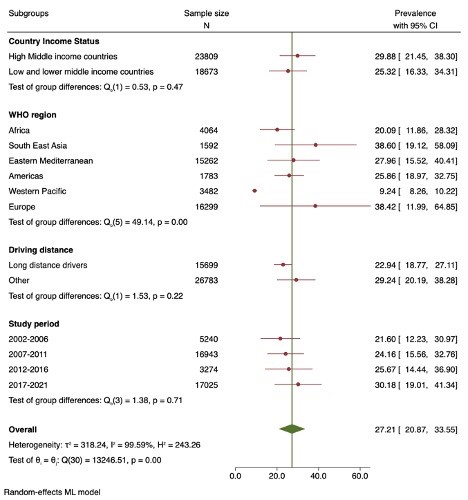

Supplement: daae087_suppl_Supplementary_Figures_4 [file daae087_suppl_supplementary_figures_4.jpeg]
