# Supplementary material for: Epidemiology of non-communicable diseases among professional drivers in LMICs: a systematic review and meta-analysis
Source: Health Promot Int. 2024 Aug 31;39(4):daae087. doi: 10.1093/heapro/daae087 (PMC11364521; doi:10.1093/heapro/daae087)
Supplement: daae087_suppl_Supplementary_Tables_2 [file daae087_suppl_supplementary_tables_2.docx]

**Supplementary Table 2: Measures used for Hypertension and DM and reported factors associated with any NCDs**

| **Author (Year)** | **Outcome assessed** | **HTN Definition criteria** | **DM and Prediabetes measure** | **Factors associated with HTN, DM, and Obesity** | | | | | | | | | | | | | | | |
| --- | --- | --- | --- | --- | --- | --- | --- | --- | --- | --- | --- | --- | --- | --- | --- | --- | --- | --- | --- |
|  |  |  |  | **Age** | **Married** | **Work duration** | **Physical inactivity** | **Alcohol use** | **Smoking** | **Overweight** | **Obesity** | **HTN** | **DM** | **High cholesterol** | **Poor dietary behaviours*** | **Using sleep inhibitors/ Sleeping < 3 hrs** | **Sitting for long duration** | **Family history (DM/CVD)** | **Others**** |
| Adedokun et al., (2017) | HTN | *≥*140/90 |  | X |  | X |  | X |  | X | X |  | X |  |  |  |  |  |  |
| Adedokun et al., (2019) | DM | *≥*140/90 | FBG | X | X |  |  |  |  |  |  | X |  |  |  |  |  |  |  |
| Amadi et al., (2018) | HTN | *≥*140/90 | FBG | X |  |  |  |  |  | X | X |  |  |  |  |  |  |  |  |
|  | DM |  |  |  |  |  |  |  |  |  | X |  |  |  |  |  |  |  |  |
| Anto et al., (2019) | HTN | *≥*140/90 |  | X |  |  | X | X | X |  |  |  |  |  | X | X | X |  |  |
|  | BMI |  |  |  |  |  | X |  |  |  |  |  |  |  | X |  |  |  |  |
| Appiah et al., (2020) | HTN + DM | *≥*130/85 | FBG |  |  |  |  |  |  |  |  |  |  |  |  |  |  |  |  |
| Borle et al., (2015) | HTN | *≥*140/90 |  |  |  |  |  |  |  |  |  |  |  |  |  |  |  |  |  |
| Devi et al., (2020) | HTN + DM | *≥*140/90 | FBG |  |  |  |  |  |  |  |  |  |  |  |  |  |  |  |  |
| Draaijer et al (2022) | HTN + DM | *≥*140/90 | FBG |  |  |  |  |  |  |  |  |  |  |  |  |  |  |  |  |
| Hachesu et al (2017) | HTN + DM | *≥*140/90 | FBG |  |  |  |  |  |  |  |  |  |  |  |  |  |  |  |  |
| Hachesu et al (2018) | HTN + DM | *≥*140/90 | FBG |  |  |  |  |  |  |  |  |  |  |  |  |  |  |  |  |
| Hayran et al (2008) | HTN | *≥*140/90 |  | X |  |  |  |  | X | X | X |  |  | X |  |  |  |  |  |
| Ibitoba et al (2022) | HTN + DM | *≥*140/90 | RBG |  |  |  |  |  |  |  |  |  |  |  |  |  |  |  |  |
| Iqbal et al (2017) | BMI |  |  |  |  |  |  |  |  |  |  |  |  |  |  |  |  |  |  |
| Jayakumar et al (2017) | HTN | *≥*140/90 |  |  |  |  |  |  |  |  |  |  |  |  |  |  |  |  |  |
| Joshi et al (2013) | HTN | *≥*140/90 |  |  |  |  |  |  |  |  |  |  |  |  |  |  |  |  |  |
| Kaewboonchoo et al (2007) | HTN | *≥*140/90 |  |  |  |  |  |  |  |  |  |  |  |  |  |  |  |  |  |
| Lalla-Edward et al (2019) | HTN + DM | *≥*140/90 | RBG |  |  |  |  |  |  |  |  |  |  |  |  |  |  |  |  |
| Loukzadeh et al (2013) | HTN + DM | *≥*140/90 | RBG |  |  |  |  |  |  |  |  |  |  |  |  |  |  |  |  |
| Marqueze et al (2013) | HTN + DM | *≥*140/90 | FBG |  |  |  |  |  |  |  |  |  |  |  |  |  |  |  |  |
| Mohebbi et al (2012) | HTN + DM | *≥*140/90 | FBG |  |  |  |  |  |  |  |  |  |  |  |  |  |  |  |  |
| Mohsen et al (2019) | HTN + DM | *≥*140/90 | RBG |  |  |  |  |  |  |  |  |  |  |  |  |  |  |  |  |
| Montazerifar et al (2019) | HTN + DM | *≥*130/85 | FBG |  |  |  |  |  |  |  |  |  |  |  |  |  |  |  |  |
| Movahed et al (2021) | HTN + DM | *≥*140/90 | FBG |  |  |  |  |  |  |  |  |  |  |  |  |  |  |  |  |
| Neralakatte et al (2021) | HTN + DM | *≥*140/90 | RBG |  |  |  |  |  |  |  |  |  |  |  |  |  |  |  |  |
| Ogbonnaya et al (2019) | HTN + DM | *≥*140/90 | RBG |  |  |  |  |  |  |  |  |  |  |  |  |  |  |  |  |
| Ozdemir et al (2009) | HTN + DM | *≥*140/90 | FBG |  |  |  |  |  |  |  |  |  |  |  |  |  |  |  |  |
| Pushpa et al (2018) | HTN | *≥*140/90 |  |  |  |  |  |  |  |  |  |  |  |  |  |  |  |  |  |
| Quichua et al (2021) | BMI |  |  |  |  |  |  |  |  |  |  |  |  |  |  |  |  |  |  |
| Ravi et al (2020) | HTN + DM | *≥*130/90 | RBG |  |  |  |  |  |  |  |  |  |  |  |  |  |  |  |  |
| Reis et al (2016) | HTN | *≥*140/90 | RBG |  |  |  |  |  |  | X | X |  |  |  |  |  |  |  |  |
|  | BMI |  |  |  |  |  | X |  |  |  |  |  |  |  |  |  |  |  |  |
| Roche et al (2021) | HTN + DM | *≥*140/90 | RBG |  |  |  |  |  |  |  |  |  |  |  |  |  |  |  |  |
| Saberi et al (2011) | HTN | *≥*140/90 |  |  |  |  |  |  |  |  |  |  |  |  |  |  |  |  |  |
| Sangaleti et al (2014) | HTN | *≥*140/90 | RBG |  |  |  |  |  |  |  | X |  |  |  |  |  |  | X |  |
|  | DM |  |  |  |  |  |  |  |  |  | X |  |  |  |  |  |  |  |  |
| Shayestefar et al (2019) | HTN + DM | *≥*140/90 | RBG |  |  |  |  |  |  |  |  |  |  |  |  |  |  |  |  |
| Showande et al (2020) | HTN + DM | *≥*140/90 | RBG |  |  |  |  |  |  |  |  |  |  |  |  |  |  |  |  |
| Siu et al (2012) | HTN + DM | *≥*130/85 | FBG | X |  |  |  |  |  |  |  |  |  |  |  |  |  | X |  |
| Smolarek et al (2013) | HTN | *≥*130/85 |  |  |  |  |  |  |  |  |  |  |  |  |  |  |  |  |  |
| Souza et al (2019) | BMI |  |  |  |  |  |  |  |  |  |  |  |  |  |  |  |  |  |  |
| Udayar et al (2014) | BMI | *≥*140/90 |  |  |  |  |  |  |  |  |  |  |  |  |  |  |  |  |  |
| Yosef et al (2020) | BMI |  |  |  |  |  |  |  |  |  |  |  |  |  |  | X | X |  | X |
| Zhidkova et al (2022) | DM and BMI |  | RBG |  |  |  |  |  |  |  |  |  |  |  |  |  |  |  |  |
| **Others: monthly income of > 220USD, family sizes of three or more members | | | | | | | | | | | | | | | | | | | |
| *Poor dietary habits: Eating late at night, high calorie intake, eating under stressful conditions. | | | | | | | | | | | | | | | | | | | |

BMI: Body Mass Index, HTN: Hypertension, DM: Diabetes Mellitus, FBG: Fasting Blood Glucose, RBG: Random Blood Glucose
