# Supplementary material for: Epidemiology of non-communicable diseases among professional drivers in LMICs: a systematic review and meta-analysis
Source: Health Promot Int. 2024 Aug 31;39(4):daae087. doi: 10.1093/heapro/daae087 (PMC11364521; doi:10.1093/heapro/daae087)
Supplement: daae087_suppl_Supplementary_Tables_1 [file daae087_suppl_supplementary_tables_1.docx]

**Supplementary Table 1: Quality assessment using the Joanna Briggs Institute (JBI) Critical Appraisal for Cross-sectional Prevalence Studies**

| JBI Qn.  Author | Was the sample frame appropriate to address the target population? | Were study participants sampled in an appropriate way? | Was the sample size adequate? | Were the study subjects and the setting described in detail? | Was the data analysis conducted with sufficient coverage of the identified sample? | Were valid methods used for the identification of the condition? | Was the condition measured in a standard, reliable way for all participants? | Was there appropriate statistical analysis? | Was the response rate adequate, and if not, was the low response rate managed appropriately? | Number of 'Yes' | Quality |
| --- | --- | --- | --- | --- | --- | --- | --- | --- | --- | --- | --- |
| Adedokun et al (2017) | Yes | Yes | Yes | Yes | Unclear | Yes | Yes | Yes | Yes | 8 | High |
| Adedokun et al (2019) | Yes | Yes | Yes | Yes | Unclear | Yes | Yes | Yes | Yes | 8 | High |
| Amadi et al (2018) | Unclear | Yes | Yes | Yes | Yes | Yes | Yes | Yes | Yes | 8 | High |
| Anto et al (2019) | Unclear | Yes | Unclear | Yes | Unclear | Yes | Yes | Yes | Yes | 6 | Moderate |
| Appiah et al (2020) | Unclear | Unclear | No | No | Yes | No | No | No | Yes | 2 | Low |
| Borle et al (2015) | Unclear | Yes | Unclear | Yes | Yes | Yes | Yes | No | Yes | 6 | Moderate |
| Devi et al (2020) | Unclear | Unclear | Unclear | Yes | Yes | Yes | No | No | Yes | 4 | Moderate |
| Draaijer et al (2022) | Yes | Yes | Yes | Yes | Yes | Yes | Yes | Yes | Yes | 9 | High |
| Hachesu et al (2017) | Unclear | Yes | Yes | No | Yes | Unclear | No | Yes | Yes | 5 | Moderate |
| Hachesu et al (2018) | Unclear | Yes | Yes | No | Yes | Unclear | No | Yes | Yes | 5 | Moderate |
| Hayran et al (2008) | Yes | Yes | Yes | Yes | Yes | Yes | Yes | Yes | Yes | 9 | High |
| Ibitoba et al (2022) | Yes | Yes | Yes | Yes | Yes | Yes | Yes | Yes | Yes | 9 | High |
| Iqbal et al (2017) | Yes | Yes | Yes | No | Yes | Yes | Yes | Yes | Yes | 8 | High |
| Jayakumar et al (2017) | Unclear | Unclear | Unclear | Yes | Yes | Yes | No | Yes | Yes | 5 | Moderate |
| Joshi et al (2013) | Yes | Yes | Yes | Yes | Yes | Yes | Unclear | Yes | Yes | 8 | High |
| Kaewboonchoo et al (2007) | Unclear | No | No | Yes | No | Yes | Yes | Yes | No | 4 | Moderate |
| Lalla-Edward et al (2019) | Yes | Yes | Unclear | Yes | Yes | Yes | Yes | Yes | Yes | 8 | High |
| Loukzadeh et al (2013) | Yes | No | Unclear | Yes | Yes | Yes | No | Yes | Yes | 6 | Moderate |
| Marqueze et al (2013) | No | No | No | Yes | Yes | Yes | Yes | Yes | Yes | 6 | Moderate |
| Mohebbi et al (2012) | Yes | Yes | Yes | Yes | Yes | Yes | Yes | Yes | Yes | 9 | High |
| Mohsen et al (2019) | Yes | Yes | Yes | No | Yes | Yes | Unclear | Yes | Yes | 7 | High |
| Montazerifar et al (2019) | Unclear | Yes | Unclear | Yes | Unclear | Yes | Yes | Yes | Yes | 6 | Moderate |
| Movahed et al (2021) | Yes | Unclear | Unclear | No | Yes | Yes | Unclear | Unclear | Yes | 4 | Moderate |
| Neralakatte et al (2021) | Yes | Unclear | Yes | No | Yes | Yes | Unclear | Yes | Yes | 6 | Moderate |
| Ogbonnaya et al (2019) | Yes | Yes | No | Yes | Yes | Yes | Yes | Yes | Yes | 8 | High |
| Ozdemir et al (2009) | Yes | Yes | Yes | Yes | Yes | Yes | Unclear | Yes | Yes | 8 | High |
| Pushpa et al (2018) | No | No | No | Yes | Yes | Yes | Yes | Yes | Yes | 6 | Moderate |
| Quichua et al (2021) | Yes | Yes | Yes | Yes | Yes | Yes | Yes | Yes | Yes | 9 | High |
| Ravi et al (2020) | Yes | Yes | Yes | Yes | Yes | Yes | Unclear | Yes | Yes | 8 | High |
| Reis et al (2016) | Unclear | Unclear | No | Yes | Yes | Yes | Unclear | Yes | Yes | 5 | Moderate |
| Roche et al (2021) | Yes | Yes | Unclear | Yes | Yes | Yes | Unclear | Yes | Yes | 7 | High |
| Saberi et al (2011) | Unclear | Unclear | Unclear | No | Yes | Yes | Unclear | Yes | Yes | 4 | Moderate |
| Sangaleti et al (2014) | Yes | Yes | Unclear | Yes | Yes | Yes | Yes | Yes | Yes | 8 | High |
| Shayestefar et al (2019) | Yes | Unclear | Unclear | Yes | Yes | Yes | Yes | Yes | Yes | 7 | High |
| Showande et al (2020) | Yes | Yes | Yes | Yes | Yes | Yes | Unclear | Yes | Yes | 8 | High |
| Siu et al (2012) | Yes | Unclear | Yes | No | Yes | Yes | Unclear | Yes | Yes | 6 | Moderate |
| Smolarek et al (2013) | No | Unclear | No | No | Yes | Yes | Unclear | No | Unclear | 2 | Low |
| Souza et al (2019) | Yes | Yes | Yes | Yes | Yes | Yes | Yes | Yes | Yes | 9 | High |
| Udayar et al (2014) | Unclear | Unclear | Unclear | Yes | Unclear | Yes | Unclear | Yes | Unclear | 3 | Low |
| Yosef et al (2020) | Yes | Yes | Yes | Yes | Yes | Yes | Yes | Yes | Yes | 9 | High |
| Zhidkova et al (2022) | Unclear | Yes | Unclear | Yes | Unclear | Yes | Unclear | Yes | Unclear | 4 | Moderate |
