# Supplementary material for: Epidemiology of non-communicable diseases among professional drivers in LMICs: a systematic review and meta-analysis
Source: Health Promot Int. 2024 Aug 31;39(4):daae087. doi: 10.1093/heapro/daae087 (PMC11364521; doi:10.1093/heapro/daae087)
Supplement: daae087_suppl_Supplementary_Tables_3 [file daae087_suppl_supplementary_tables_3.docx]

| **Supplementary Table 3: Recommended interventions (N=42)** | | |
| --- | --- | --- |
| **Intervention** | **n** | **%** |
| Health promotion (including Education programs on life style modification and public awareness) | 23 | 54.8 |
|  |  |  |
| Regular screening for noncommunicable diseases and the risk factors | 21 | 50.0 |
|  |  |  |
| Improvement of policies governing the transportation sectors (including driving hours, and overall organisation of work) | 5 | 11.9 |
|  |  |  |
| Establishment (provision) of facilities for physical exercise and recreation. | 8 | 19.1 |
